# Leisure Sedentary Behavior and Risk of Lung Cancer: A Two-Sample Mendelian Randomization Study and Mediation Analysis

**DOI:** 10.3389/fgene.2021.763626

**Published:** 2021-10-28

**Authors:** Yan Gao, Jiarui Mi, Zhengye Liu, Qibin Song

**Affiliations:** ^1^ Department of Oncology, Cancer Center, Remin Hospital of Wuhan University, Wuhan, China; ^2^ Master Program of Biomedicine, Karolinska Institutet, Solna, Sweden; ^3^ Department of Orthopedics, Zhongnan Hospital of Wuhan University, Wuhan, China

**Keywords:** mendelian randomisation, leisure sedentary behaviors, television watching, lung cancer, smoking behavior

## Abstract

Leisure sedentary behavior, especially television watching, has been previously reported as associated with the risk of lung cancer in observational studies. This study aims to evaluate the causal association with two-sample Mendelian randomization (MR) analysis. Single nucleotide polymorphisms associated with leisure television watching, computer use, and driving were extracted from genome-wide association studies. Summary-level results of lung cancer overall and histological types were obtained from International Lung Cancer Consortium (ILCCO). In univariable MR using inverse-variance-weighted method, we observed causal effects of television watching on lung cancer [OR, 1.89, 95% confidence interval (CI), 1.41, 2.54; *p* = 2.33 × 10^−5^], and squamous cell lung cancer (OR, 2.37, 95% CI, 1.58, 3.55; *p* = 3.02 × 10^−5^), but not on lung adenocarcinoma (OR, 1.40, 95% CI, 0.94, 2.09; *p* = 0.100). No causal effects of computer use and driving on lung cancer were observed. Television watching significantly increased the exposure to several common risk factors of lung cancer. The associations of television watching with lung cancer and squamous cell lung cancer were compromised after adjusting for smoking quantity with multivariable MR. Our mediation analyses estimated indirect effects of television watching on lung cancer (beta, 0.31, 95% CI, 0.13, 0.52; *p* = 6.64 × 10^−4^) and squamous cell lung cancer (beta, 0.33, 95% CI, 0.14, 0.53, *p* = 4.76 × 10^−4^) mediated by smoking quantity. Our findings indicate that television watching is positively correlated with the risk of lung cancer, potentially mediated through affecting smoking quantity.

## Introduction

Nowadays people are more and more prone to extended sedentary behaviors in their daily life due to the increased time spent on television watching, computer use, and driving (Healy et al., 2011; Proper et al., 2011). Previous scientific evidence has shown that increased leisure sedentary behaviors are associated with a higher risk of all-cause mortality, incidence, and mortality of the cardiovascular disease, type 2 diabetes, and cancer (Physical Activity Guidelines Advisory Committee, 2018). However, the evidence that indicates the relationship between sedentary behavior and cancer has been graded as moderate, and there is limited evidence that suggests a direct link between sedentary behavior and incident lung cancer (Physical Activity Guidelines Advisory Committee, 2018). Several publications have studied the associations between leisure sedentary behavior and lung cancer (Schmid and Leitzmann, 2014; Shen et al., 2014). While the pieces of evidence are all based on observational studies, they may not rule out the possibility of biases including confounding factors and reverse causality. Besides, randomized controlled studies are unethical and inappropriate to be performed on this topic.

Mendelian randomization is a method that uses genetic variants as proxies for exposures. Since single nucleotide polymorphisms (SNPs) are assigned randomly at conception, thus are unlikely to be affected by lifestyle and environmental factors (Davey Smith and Hemani, 2014). This feature of MR diminishes the risks of confounding factors and reverse causality which are common in traditional observational studies (Smith and Ebrahim, 2003). Multivariable Mendelian randomization (MVMR) is a newly developed extension to MR which enables us to assess several exposures simultaneously and estimate causal effects of the exposures after adjusting for the others (Sanderson et al., 2020).

In this study, we estimated the effects of three leisure sedentary behaviors (leisure television watching, leisure computer use, driving) on the risk of lung cancer and also its subtypes with MR analyses. We further assess the effects of television watching on several common risk factors of lung cancer and estimates the causal inference after adjusting for different factors with MVMR. In addition, we also calculated the proportion of television watching’s effect on lung cancer mediated by smoking quantity with a mediation analysis (Burgess and Thompson, 2015; Burgess et al., 2017).

## Materials and Methods

### Study Design

In this study, we have used instrumental variables for leisure sedentary behaviors (including leisure television watching, leisure computer use, and driving) from a previous publication, and investigated the causal effects of leisure sedentary behaviors on the risk of lung cancer by using two-sample MR analyses (van de Vegte et al., 2020). In addition, we also assessed the effect of leisure television watching on common risk factors of cancer including smoking, body mass index (BMI), triglycerides, and total cholesterol. We performed a mediation analysis to estimate the proportion of television watching’s effect on lung cancer mediated through smoking behavior.

### Data Source

Instrumental variables for leisure sedentary behaviors were obtained from summary-level GWAS results of a previous publication based on the UK biobank (*n* = 422218; European ancestry) (van de Vegte et al., 2020). The reported average daily time spent on television watching, computer use, and driving was 2.8 h (SD 1.5), 1.0 h (SD 1.2), and 0.9 h (SD 1.0). 209, 35, and 4 SNPs were identified as significantly associated with television watching, computer use, and driving respectively (*p* < 1 × 10^−8^). We further clumped the SNPs in linkage disequilibrium (LD, R2 ≥ 0.001 or within 5000 kb). PhenoScanner V2 database has been used for detecting potential pleiotropy of all the included SNPs (http://www.phenoscanner.medschl.cam.ac.uk/) (Staley et al., 2016). SNPs that were identified as associated with potential confounders (BMI, obesity, alcohol consumption, smoking) were excluded from the analyses. The strength of the included SNPs was assessed with R2 and F-statistics (Supplementary Table S1) (Physical Activity Guidelines Advisory Committee, 2018). The risk of weak instrument bias is relatively low with an F-statistic over 10 (Palmer et al., 2012). The included SNPs had substantial strength with F-statistics ranging from 29 to 101 for television watching, 32 to 84 for computer use, and 41 to 46 for driving (Supplementary Table S4).

Summary-level GWAS results of lung cancer, lung adenocarcinoma, and Squamous cell lung cancer were obtained from a meta-analysis from the International Lung Cancer Consortium (ILCCO) conducted in a non-overlapping population (11 348 lung cancer cases and 15 861 controls; European ancestry) (Wang et al., 2014). Detailed information of all data sources is shown in Supplementary Table S1.

### Statistical Analysis

Several MR analyses methods were used in this study to estimate the causal effect of leisure sedentary behaviors on lung cancer. The inverse-variance weighted (IVW) method was conducted as the main analysis of MR to combine the Wald ratio of individual SNPs. Heterogeneity of the analyses was assessed with Cochran’s Q values, I^2^ statistics, and the H statistics (Higgins et al., 2003; Physical Activity Guidelines Advisory Committee, 2018). We performed random effect IVW when significant heterogeneity was detected (p of Cochran’s Q < 0.1) otherwise fixed effect IVW was used. Other statistical tests including MR-Egger, weighted median, MR-PRESSO, MR-APSS, and constrained maximum likelihood and model averaging (cML-MA) have been used as sensitivity tests (Bowden et al., 2016; Burgess and Thompson, 2017; Physical Activity Guidelines Advisory Committee, 2018; Hu et al., 2021). The MR-Egger method identifies and corrects for potential pleiotropy while the weighted median method can provide consistent causal estimates when up to fifty percent of the weight of the analyses were from invalid instrumental variables (Bowden et al., 2016; Burgess and Thompson, 2017). MR-PRESSO analyses were performed to identify and correct for potential outliers which also help to avoid potential horizontal pleiotropy (Verbanck et al., 2018). MR-APSS is a newly developed approach to MR which uses the genome-wide summary data to build a background model. The method is capable of telling whether a SNP with moderate effect belongs to background or foreground component, thus includes more SNPs to improve statistical powers (Hu et al., 2021). cML-MA is a MR method that is, robust to invalid instrumental variables and also uncorrelated or correlated pleiotropy (Xue et al., 2021). We have also made different diagnostic plots to describe the robustness of the causal estimates of the MR analyses (Physical Activity Guidelines Advisory Committee, 2018). The scatter plots present the relationship of SNPs-exposure association against SNPs-outcome associations, while the forest plots visualize the contribution of individual instrumental variables to the overall causal estimation. Leave-one-out plots were used to visualize the results from leave-one-out analyses which recalculate the causal estimates from IVW by dropping out one SNP at a time to verify if the estimates were biased or driven by an outlier. The power of the analyses was calculated with an online tool (https://shiny.cnsgenomics.com/mRnd/) (Brion et al., 2013).

To investigate the potential mechanisms through which genetically proxied leisure television watching affects the risk of lung cancer, we also calculated its effect on common risk factors of lung cancer including smoking status (age of smoking initiation; cigarettes smoked per day; smoking initiation, smoking cessation), BMI, triglycerides and total cholesterol (Willer et al., 2013; Locke et al., 2015; Liu et al., 2019). Summary level results of these potential mediators were retrieved from GWAS results based on populations not overlapping with the exposures. Detailed information of their data sources was presented in Supplementary Table S1. The direct effects of television watching on lung cancer after adjusting for the risk factors were estimated with MVMR (Sanderson et al., 2020). Finally, we assessed the indirect effect of television watching on lung cancer *via* smoking quantity with the method as previously described (Burgess and Thompson, 2015; Burgess et al., 2017).

All statistical analyses were two-sided. A Bonferroni adjusted *p*-value below 0.0056 (0.05/9) was considered significant and indicated strong evidence of associations. All analyses were conducted with R (version 4.0.2), TwoSampleMR (0.5.5), Mendelian Randomization (0.5.0), and MR-PRESSO packages (Yavorska and Burgess, 2017; Hemani et al., 2018; Verbanck et al., 2018).

## Results

### Leisure Sedentary Behaviors and Lung Cancer

**FIGURE 1:**
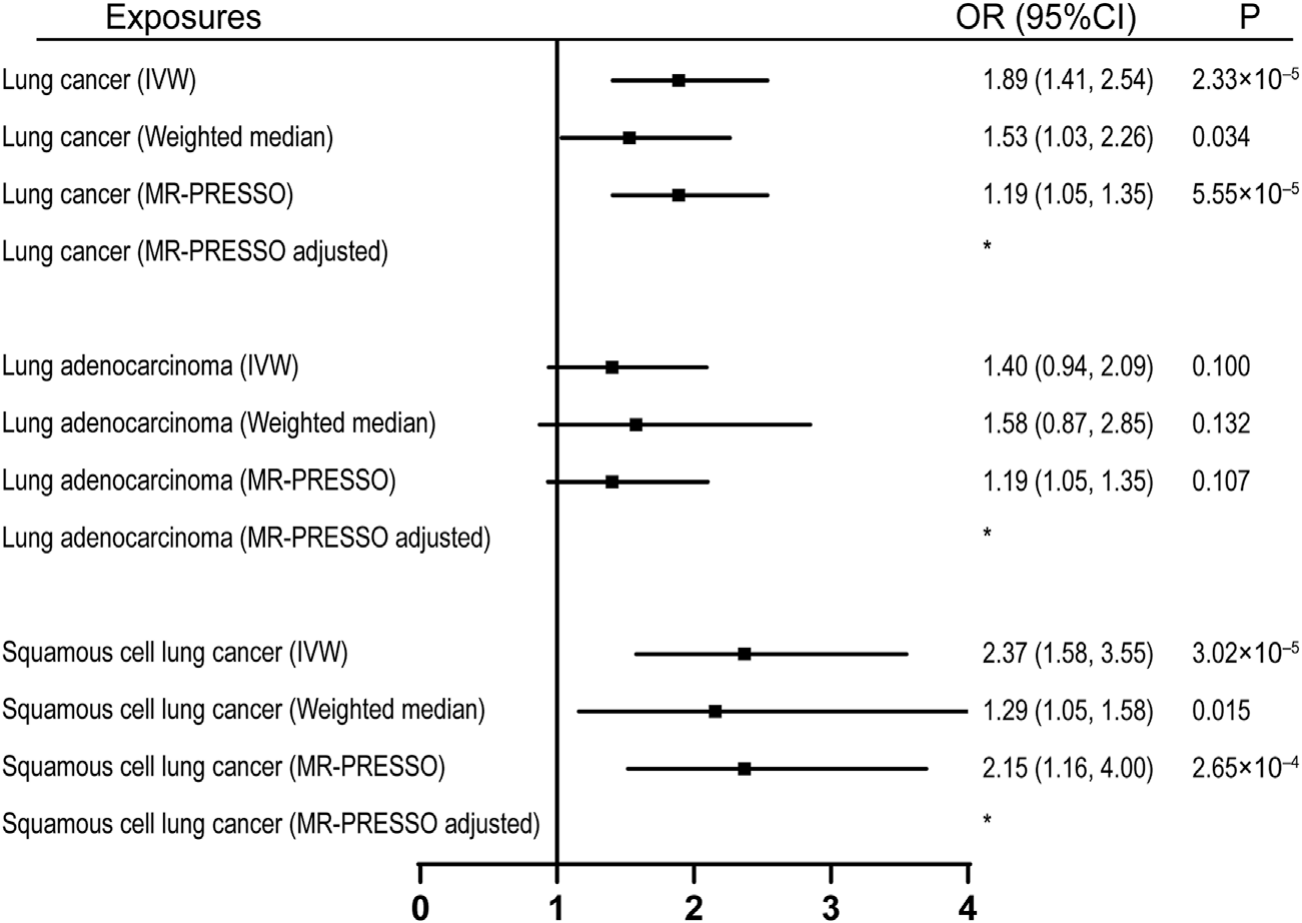
Univariable MR analyses showing the causal effects of genetically proxied leisure television watching on overall lung cancer and different histological types with IVW, weighted median, and MR-PRESSO methods. *No outlier detected. IVW, inverse-variance weighted; OR, odds ratio; CI, confidence interval; MR, Mendelian randomization.

92 SNPs have been used to genetically proxy the effect of leisure television watching (Supplementary Table S4). Television watching was positively correlated with the risk of overall lung cancer, and the risk of squamous cell lung cancer, but not with the risk of lung adenocarcinoma (Figure 1). The odds ratios (ORs) were 1.89 [95% confidence interval (CI), 1.41, 2.54; *p* = 2.33 × 10^−5^], 1.40 (95% CI, 0.94, 2.09; *p* = 0.100), and 2.37 (95% CI, 1.58, 3.55; *p* = 3.02 × 10^−5^) for lung cancer, lung adenocarcinoma, and squamous cell lung cancer respectively for 1 SD increase of television watching time (1.5 h) by using a IVW method (Figure 1). We have high power to detect the effect of television watching on lung cancer (99% power to detect an OR of 1.89, minimal and maximal detectable OR with 80% power: 1.51/0.70) and squamous cell lung cancer (100% power to detect an OR of 2.37, minimal and maximal detectable OR with 80% power: 1.55/0.56). But lower in detecting the effect on lung adenocarcinoma (56% power to detect an OR of 1.40, minimal and maximal detectable OR with 80% power: 1.54/0.57) (Table 1). The intercepts’ *p* values were >0.05 with the MR-Egger pleiotropy test, indicating no pleiotropic bias exists in assessing the effect of leisure television watching with the IVW method (Table 2). The causal results remained consistent with the MR-PRESSO method, and no outliers were identified (Figure 1). Leave-one-out analysis showed that the associations of television watching with the outcomes were not driven by any single SNP (Supplementary Figures S3, S15, S27). The associations of individual SNPs with exposures and outcomes were presented in Supplementary Table S4. With MR-APSS, the effect of television watching on lung cancer remained constant (OR = 1.61, 95% CI, 1.10, 2.37, *p* = 0.015, Supplementary Figure S37), while no significant causal effect was observed on the histological subtypes (Supplementary Figures S38, S39). By using cML-MA, time spent on television showed a positive correlation with lung cancer (OR = 1.95, 95% CI, 1.49, 2.56, *p* = 1.20 × 10^−6^) and squamous cell lung cancer (OR = 2.49, 95% CI, 1.64, 3.78, *p* = 1.93 × 10^−5^), but not with lung adenocarcinoma (OR = 1.40, 95% CI, 0.93, 2.10, *p* = 0.109).

**TABLE 1:** Statistical power calculation for the univariable Mendelian randomization analyses (two-sided, *α* = 0.05).

Exposures	Outcomes	Sample size	K^a^	*R* ^2^ (%)	Statistical power^b^	Minimal/maximal detectable OR^c^
Television watching	Lung cancer	27209	0.715466	1.00	0.99	1.51/0.70
Television watching	Lung adenocarcinoma	18336	0.2311	1.00	0.56	1.54/0.57
Television watching	Squamous cell lung cancer	18313	0.217782	1.00	1.00	1.55/0.56
computer use	Lung cancer	27209	0.715466	0.23	0.12	2.57/0.51
computer use	Lung adenocarcinoma	18336	0.2311	0.23	0.15	2.20/0.22
computer use	Squamous cell lung cancer	18313	0.217782	0.23	0.10	2.22/0.20
Driving	Lung cancer	27209	0.715466	0.03	0.07	NA/0.26
Driving	Lung adenocarcinoma	18336	0.2311	0.03	0.05	4.09/NA
Driving	Squamous cell lung cancer	18313	0.217782	0.03	0.05	4.09/NA

a Proportions of cases in the outcomes.

b The statistical power to detect the associations in the Mendelian randomization analyses.

c The maximal and minimal detectable odds ratios with power over 0.8 at a significance level of 0.05.

**TABLE 2:** MR-Egger pleiotropic test of the causal associations between leisure sedentary behavior and risk of lung cancer.

Exposures	Outcomes	MR-egger		Pleiotropy test		
		OR (95% CI)	*p*-value	Intercept	SE (intercept)	*p*-value
Television watching	Lung cancer	1.63 (0.31, 8.47)	0.564	0.002	0.013	0.858
Television watching	Lung adenocarcinoma	1.52 (0.16, 14.31)	0.715	−0.001	0.018	0.942
Television watching	Squamous cell lung cancer	1.02 (0.09, 12.21)	0.987	0.014	0.020	0.501
Computer use	Lung cancer	0.37 (0.05, 2.85)	0.342	−0.035	0.035	0.324
Computer use	Lung adenocarcinoma	1.18 (0.61, 2.26)	0.622	−0.031	0.054	0.584
Computer use	Squamous cell lung cancer	0.84 (0.39, 1.79)	0.648	−0.028	0.057	0.626
Driving	Lung cancer	1.02 (0.32, 3.22)	0.972	0.031	0.043	0.601
Driving	Lung adenocarcinoma	1.34 (0.48, 3.75)	0.578	−0.049	0.140	0.787
Driving	Squamous cell lung cancer	0.62 (0.02, 18.61)	0.781	−0.083	0.343	0.849

**FIGURE 2:**
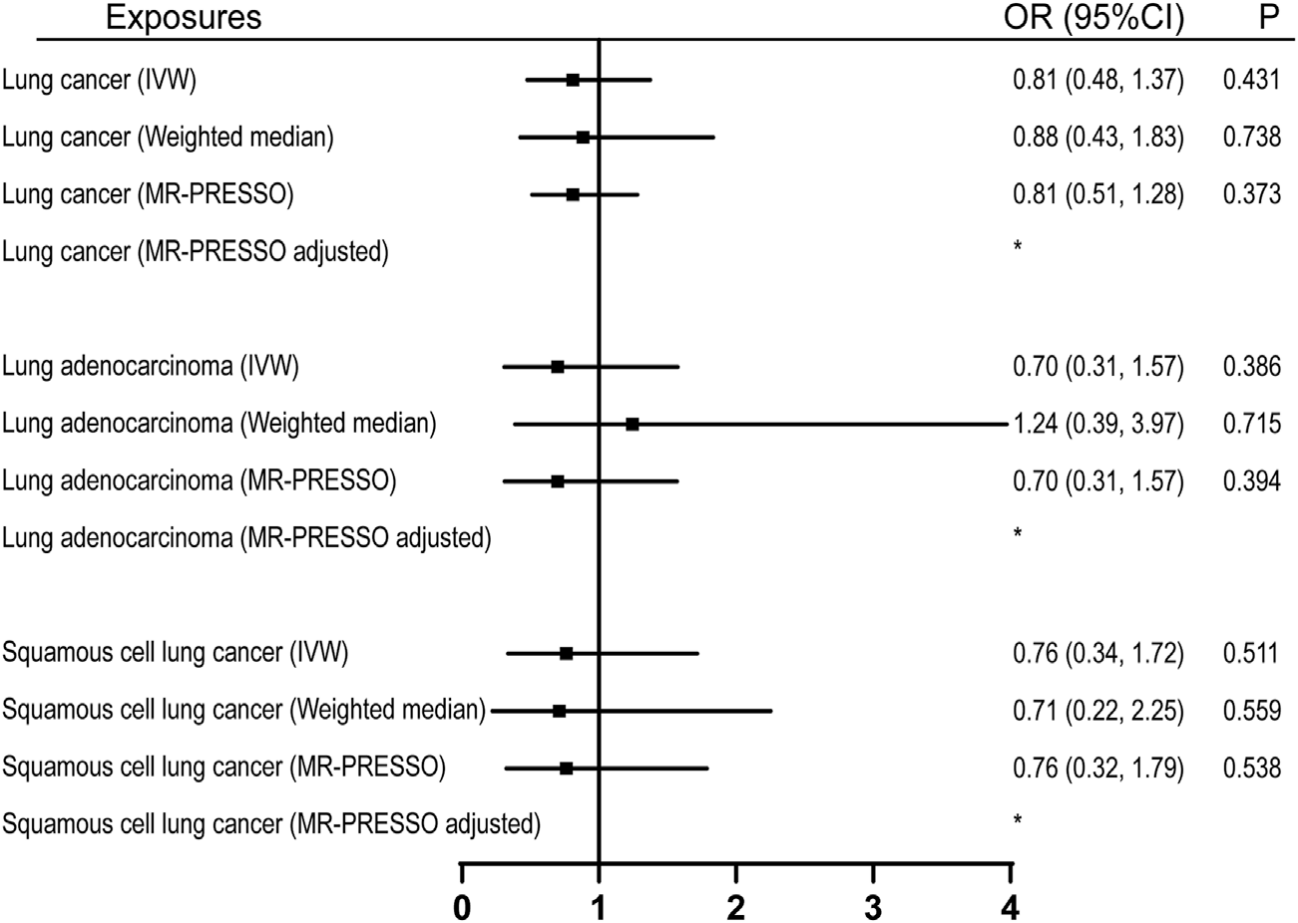
Univariable MR analyses showing the causal effects of genetically proxied leisure computer use on overall lung cancer and different histological types with IVW, weighted median, and MR-PRESSO methods. *No outlier detected. IVW, inverse-variance weighted; OR, odds ratio; CI, confidence interval; MR, Mendelian randomization.

22 were available as instrumental variables in assessing the effect of leisure computer use (Supplementary Table S4). No causal effect of computer use was observed, however, with limited power (Figure 2; Table 1). No pleiotropy, no heterogeneity, and no outliers were identified in the analyses (Tables 2, 3, and Figure 2). 3 SNPs were selected as instrumental variables for driving. We did not observe a causal effect of driving on lung cancer (Supplementary Table S2).

**TABLE 3:** Heterogeneity test of the Mendelian randomization analyses between leisure sedentary behavior and risk of lung cancer.

Exposures	Outcomes	Cochrane’s Q	Qdf^a^	*p*-value	I^2^ (%)	H statistic (95% CI)
Television watching	Lung cancer	114.88	91	0.046	21	1.124 (0.983, 1.285)
Television watching	Lung adenocarcinoma	92.76	91	0.429	2	1.010 (0.902, 1.130)
Television watching	Squamous cell lung cancer	108.60	90	0.089	17	1.098 (0.952, 1.268)
Computer use	Lung cancer	15.70	21	0.786	0	0.865 (0.624, 1.179)
Computer use	Lung adenocarcinoma	20.80	21	0.471	0	0.995 (0.730, 1.357)
Computer use	Squamous cell lung cancer	23.15	21	0.336	9	1.050 (0.828, 1.332)
Driving	Lung cancer	1.02	2	0.600	0	0.715 (0.231, 2.216)
Driving	Lung adenocarcinoma	0.45	2	0.799	0	0.473 (0.153, 1.467)
Driving	Squamous cell lung cancer	6.56	2	0.038	70	1.812 (0.979, 3.352)

a Degree of freedom of Cochrane’s Q.

### Television Watching and Risk Factors of Lung Cancer

**FIGURE 3:**
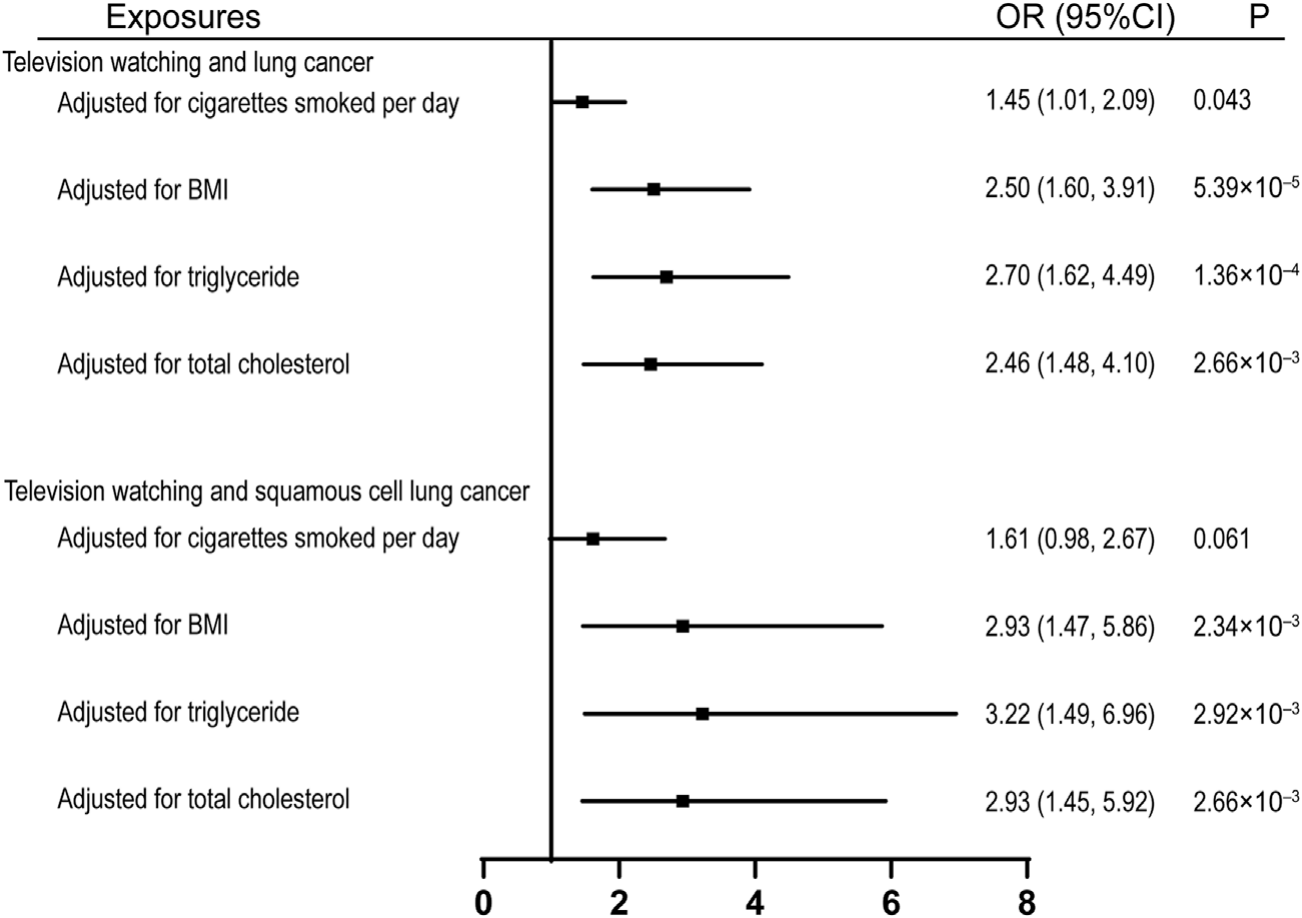
Multivariable MR analyses showing the associations of genetically proxied leisure television watching with overall lung cancer and squamous cell lung cancer after adjusting for common risk factors of lung cancer. BMI, body mass index; OR, odds ratio; CI, confidence interval; MR, Mendelian randomization.

To investigate the potential mechanisms through which leisure television watching affects lung cancers, we assessed its effect on several common risk factors of lung cancer. Genetically proxied television watching was found to be significantly associated with the age of smoking initiation, cigarettes smoked per day, smoking initiation, smoking cessation, BMI, and triglycerides, but not with total cholesterol (Supplementary Table S3). we also estimated the causal effects of television watching on lung cancer and squamous cell lung cancer after adjusting for several common risk factors and observed that the effects were compromised after the adjustment for the quantity of smoking, with ORs equal to 1.45 (95% CI, 1.01, 2.09; *p* = 0.043) and 1.61 (95% CI, 1.61, 2.67; *p* = 0.061) respectively (Figure 3). However, the effects remained significant after adjusting for the effects of BMI, total triglyceride, and total cholesterol (Figure 3).

### Mediation Analysis

Due to the high risk of cigarette smoking on lung cancers especially squamous cell lung cancer, we performed a mediation analysis to estimate the proportion of leisure television watching’s effect on lung cancers mediated through the quantity of smoking. The mediation effect of cigarette smoked per day in the causal pathway from television watching to lung cancer was 0.31 (95% CI, 0.13, 0.52; *p* = 6.64 × 10^−4^), accounting for 51.8% (95% CI, 19.2%, 104.0%) of the total effect (Table 4). The mediation effect was 0.33 (95% CI, 0.14, 0.53, *p* = 4.76 × 10^−4^) in the pathway from television watching to squamous cell lung cancer with a mediation proportion of 41.6% (95% CI, 15.2%, 88.9%) (Table 4).

**TABLE 4:** Mediation effect of television watching on lung cancer and squamous cell lung cancer *via* smoking quantity.

Exposure	Mediator	Outcomes	Total effect^a^ Effect X^b^ Effect M^c^ Mediation effect^d^ Mediated proportion					
			Effect size (95% CI)	Effect size (95% CI)	Effect size (95% CI)	Effect size (95% CI)	*p* values	(%) (95% CI)
Television watching	Cigarettes per day	Lung cancer	0.64 (0.34, 0.93)	0.12 (0.06, 0.19)	2.50 (1.76, 3.25)	0.31 (0.13, 0.52)	0.00066435	51.8% (19.2%, 104.0%)
Television watching	Cigarettes per day	Squamous cell lung cancer	0.86 (0.42, 1.31)	0.12 (0.06, 0.19)	2.67 (2.06, 3.27)	0.33 (0.14, 0.53)	0.00047622	41.6% (15.2%, 88.9%)

a Total effect: the effect of exposures on outcomes.

b Effect X: the effect of exposures on mediators.

c Effect M: the effect of mediators on outcomes.

d Mediation effect: the indirect effect of exposures on outcomes *via* mediators.

## Discussion

In this study, we analyzed the causal effect of leisure sedentary behaviors on two main histologic subtypes of lung cancer utilizing MR analyses. We found that more time spent on television viewing is significantly associated with an increased risk of lung cancer, especially the risk of squamous cell lung cancer, while no causal effect of computer use and driving was observed with relatively low power for the analyses of these two exposures. Our further mediation analysis indicated that this association is possibly mediated by increasing the consumption of cigarettes.

Sedentary behavior is emerging as an independent risk factor for multiple chronic diseases, including cardiovascular disorders, and cancer. Several studies have reported that compared to sedentary professions, occupations that require walking, or laboring had lower risk of lung cancer (Bak et al., 2005; Steindorf et al., 2006). Television viewing time has been reported as the most important leisure-time associated sedentary behavior and has been mostly studied (Clark et al., 2011; Lam et al., 2013). It is suggested that television watching time better captures the aspect of sedentary behavior that is, relevant to cancer than other sedentary behaviors (Schmid and Leitzmann, 2014). TV viewing is often associated with increased calorie intake and an increased chance of smoking (Gidwani et al., 2002; Wiecha et al., 2006). Indeed, our study verified the causal association between television watching and the risk of lung cancer, but not with leisure computer use nor driving. Our results are in line with previous studies which reported that prolonged television watching time increased risks of several types of cancer (Patel et al., 2006; Howard et al., 2008; Ukawa et al., 2013; Schmid and Leitzmann, 2014). A meta-analysis including 43 observational studies found that television watching is associated with lung cancer, colon cancer, and endometrial cancer, but not with other types of cancer (Schmid and Leitzmann, 2014). In a large-scale cohort study enrolling 54,258 cancer-free adults, the hazard ratio of lung cancer for males that spent more than 4 h per day on television watching was 1.36 (95% CI 1.04–1.80) compared to less than 2 h per day (Ukawa et al., 2013).

Several potential mechanisms have been proposed as potential mediators for the association between television watching and cancer. Prolonged sedentary behaviors are shown to increase the level of multiple inflammatory factors including tumor-necrosis factor-alpha, interleukin-6, and leptin that may contribute to the development of lung cancer (Shih et al., 2006; Terzidis et al., 2009; van Kruijsdijk et al., 2009; Zhan et al., 2009). Gain of body weight and obesity due to television watching can facilitate carcinogenesis through multiple pathways (Calle and Kaaks, 2004; Renehan et al., 2008). Compared to occupational sedentary behaviors, television watching was found to have a strong association with the biomarkers of cardiometabolic disorders (Hu et al., 2003). Increased smoking initiation and smoking quantity are also important factors that accompany television watching behavior (Gidwani et al., 2002). Smoking is known to be associated with the risk of several different types of cancer (Botteri et al., 2008; Gandini et al., 2008). It is reported that people viewing less television had a greater lung cancer-free life expectancy of more than 1 year (Cuthbertson et al., 2020). In this study, we estimated the causal effects of television watching on lung cancer after adjusting for several common risk factors and observed that the effects were compromised after adjusting for cigarettes smoked per day. However, television watching was still significantly correlated with the risk of overall lung cancer and squamous cell lung cancer after adjusting for BMI, total triglyceride, and total cholesterol. Our results indicate that smoking behavior may be the major mediator in the causal pathway from television watching to lung cancer. Furthermore, we have used mediation analyses to calculate the proportion of indirect effect that smoking mediated and observed that a large proportion of television watching’s effect was mediated by smoking quantity (Table 4). The observed mediation effects are in line with our findings from univariable MR that television watching is causally associated with lung cancer overall and squamous cell lung cancer but not lung adenocarcinoma since the influence of smoking on adenocarcinoma is not as great as on other histological subtypes (Kabat, 1996).

Our study has several strengths. This study was the first MR analysis concerning television watching and the main histologic subtypes of lung cancer, including adenocarcinoma and squamous carcinoma. Besides, by using SNPs as instrumental variables of leisure sedentary behaviors, the MR study reduced the risk of reverse causality and confounding factors which are commonly seen in observational studies. Furthermore, the data source of the included exposures and outcomes were obtained from the two largest GWAS to date from two non-overlapping samples. We also confined this study to the European population to reduce population stratification.

However, there were still some limitations in this study. First, due to the limited number of SNPs available and low *R* ^2^ for computer use and driving, the power of analyses for these two traits was relatively low. Second, the generalizability of the conclusion was restricted since the used GWAS data were mainly based on the European population, it is necessary to validate the conclusions in other populations. Third, the utilization of genetic IVs represented that the effects of the exposures to the outcomes were lifelong, which can be different from the actual situation. Lastly, the associations between television watching and lung cancer were found to be partly mediated through smoking quantity. However, other mediators that we haven’t studied may also exist which requires further study.

In summary, our MR study provided significant evidence for the positive association of television watching with the risk of overall lung cancer and squamous cell lung cancer, while the effects were to a large proportion mediated through enhancing the smoking behavior. Reducing time spent watching television especially in smoking population may be a beneficial measure in preventing lung cancer.

## Data Availability

The original contributions presented in the study are included in the article/Supplementary Material, further inquiries can be directed to the corresponding authors.
