# Supplementary figures and images for: Leisure Sedentary Behavior and Risk of Lung Cancer: A Two-Sample Mendelian Randomization Study and Mediation Analysis
Source: Front Genet. 2021 Oct 28;12:763626. doi: 10.3389/fgene.2021.763626 (PMC8582637; doi:10.3389/fgene.2021.763626)

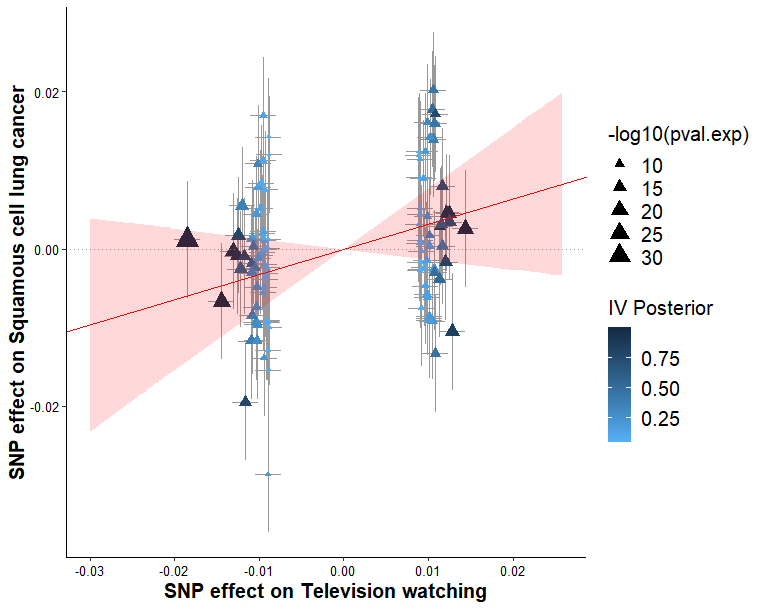

Supplement: Supplementary file 1 [file Image3.TIFF]

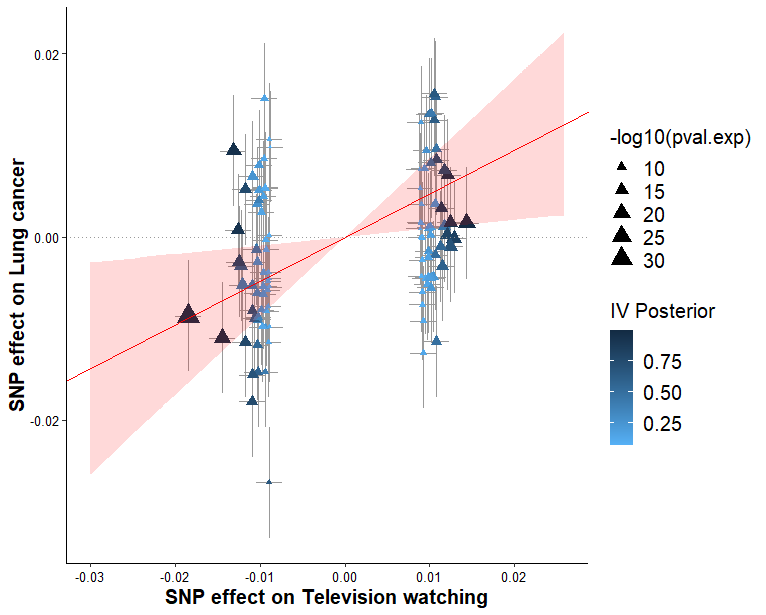

Supplement: Supplementary file 3 [file Image1.TIFF]

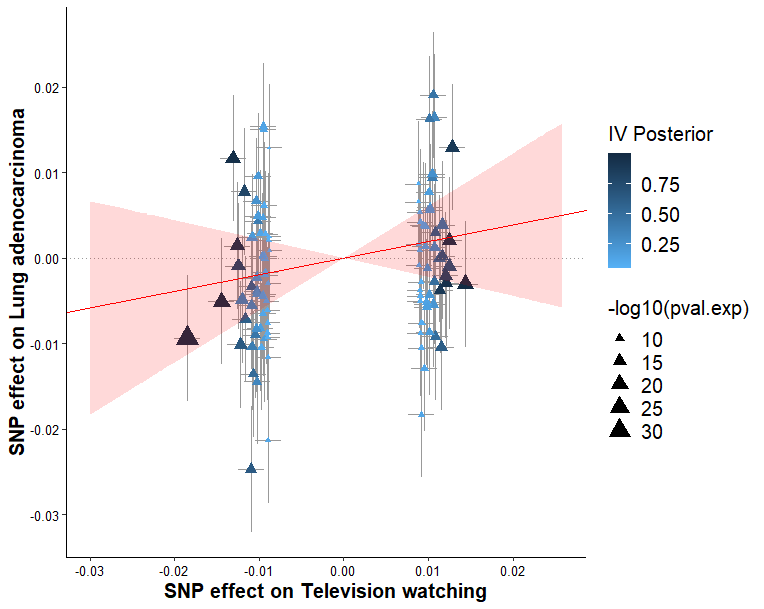

Supplement: Supplementary file 9 [file Image2.TIFF]
