# Supplementary material for: Leisure Sedentary Behavior and Risk of Lung Cancer: A Two-Sample Mendelian Randomization Study and Mediation Analysis
Source: Front Genet. 2021 Oct 28;12:763626. doi: 10.3389/fgene.2021.763626 (PMC8582637; doi:10.3389/fgene.2021.763626)

Supplementary Figure 25. Scatter plots presenting the relationship between television watching and squamous cell lung cancer.


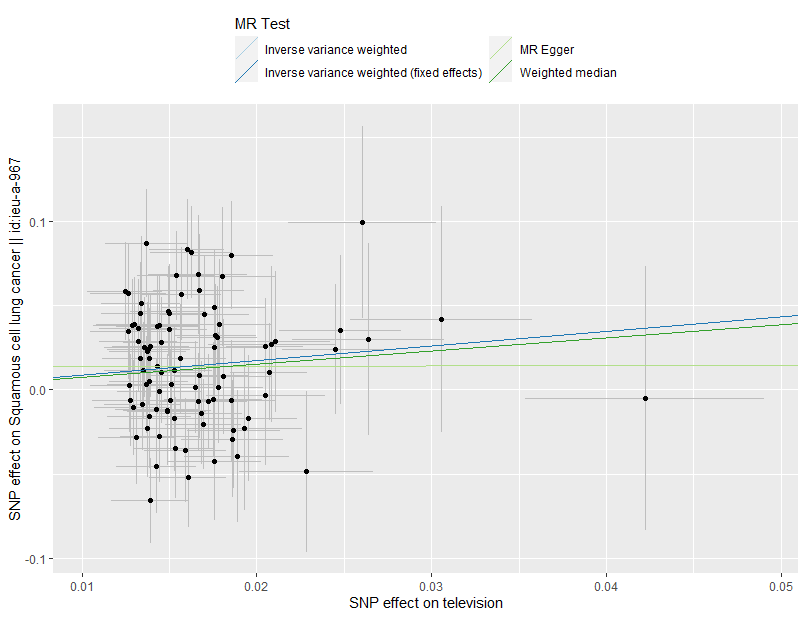


Supplementary Figure 26. Forest plots presenting the relationship between television watching and squamous cell lung cancer.


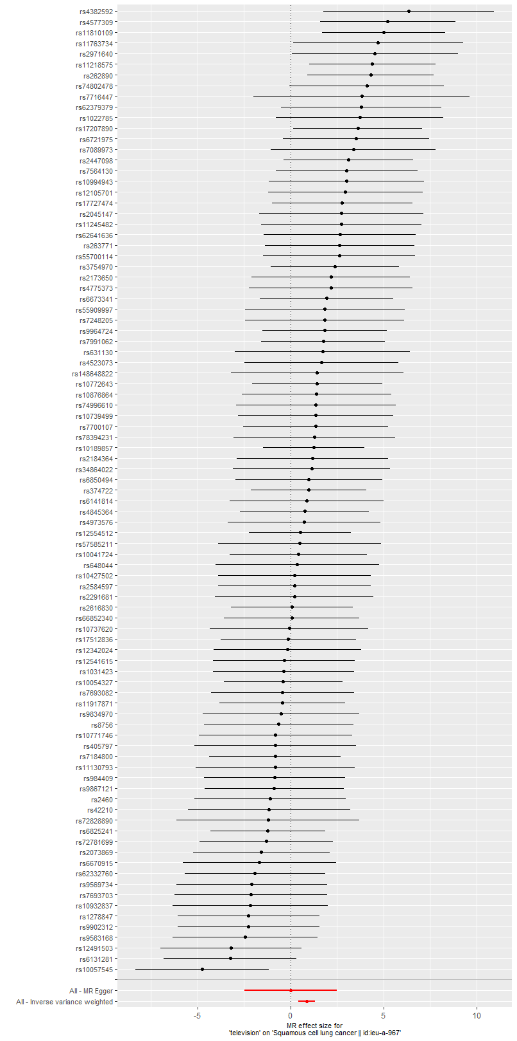


Supplementary Figure 27. Leave-one-out plot presenting the relationship between television watching and squamous cell lung cancer.


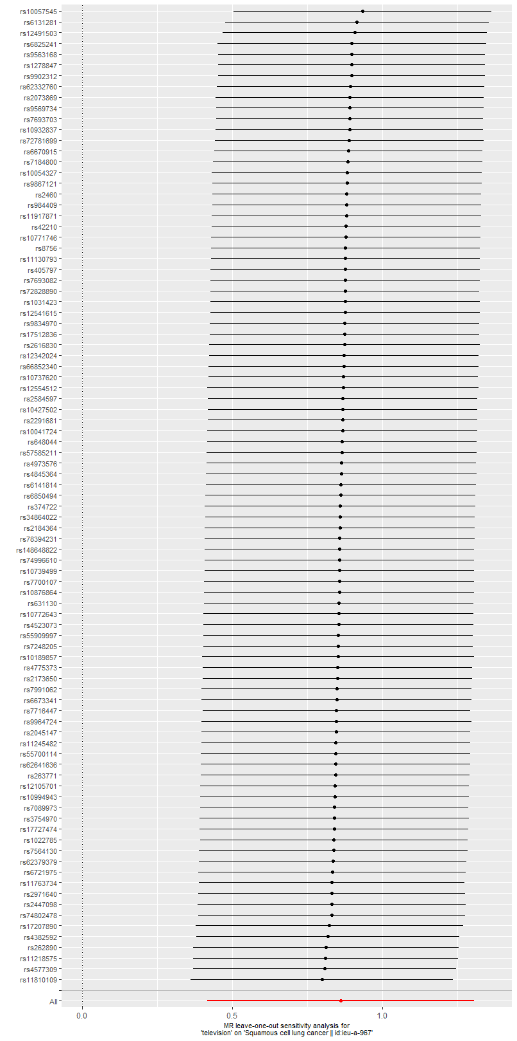


Supplementary Figure 28. Funnel plots presenting the relationship between television watching and squamous cell lung cancer.


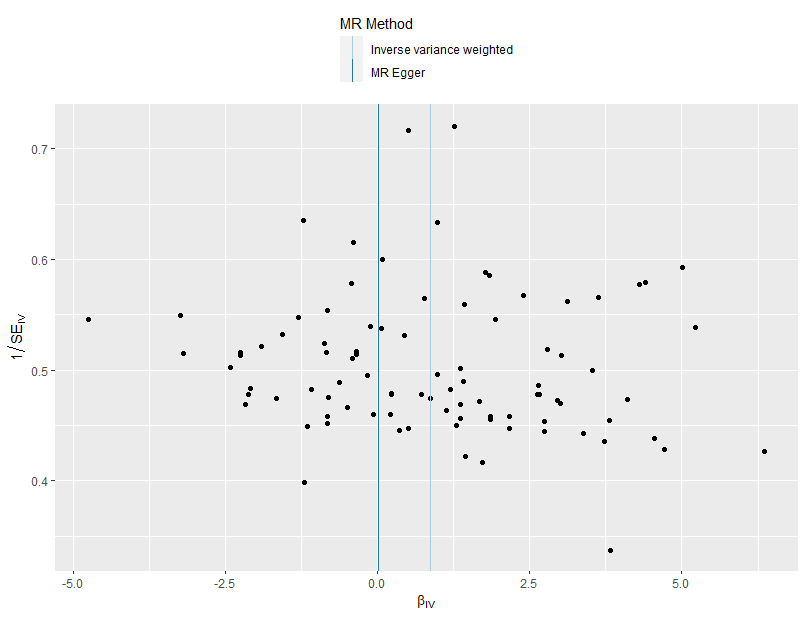


Supplementary Figure 29. Scatter plot presenting the relationship between computer use and squamous cell lung cancer.


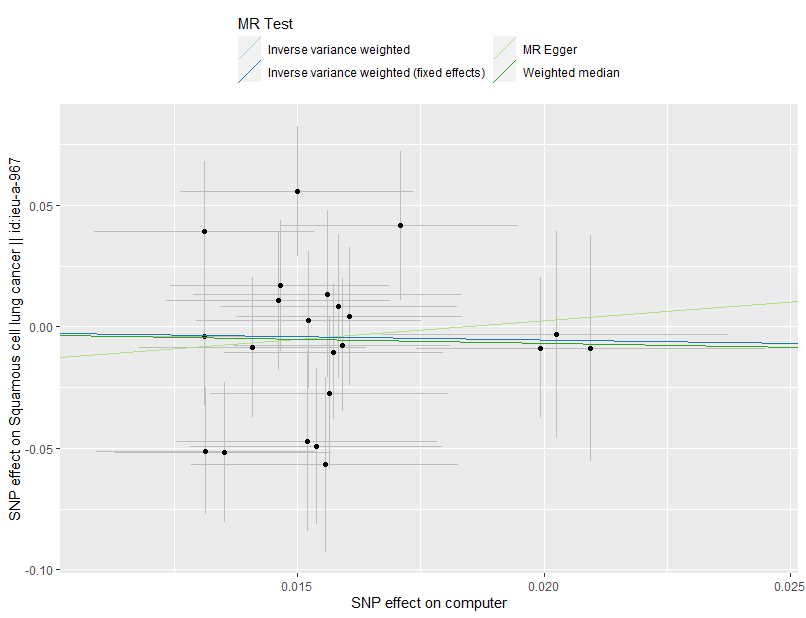


Supplementary Figure 30. Forest plot presenting the relationship between computer use and squamous cell lung cancer.


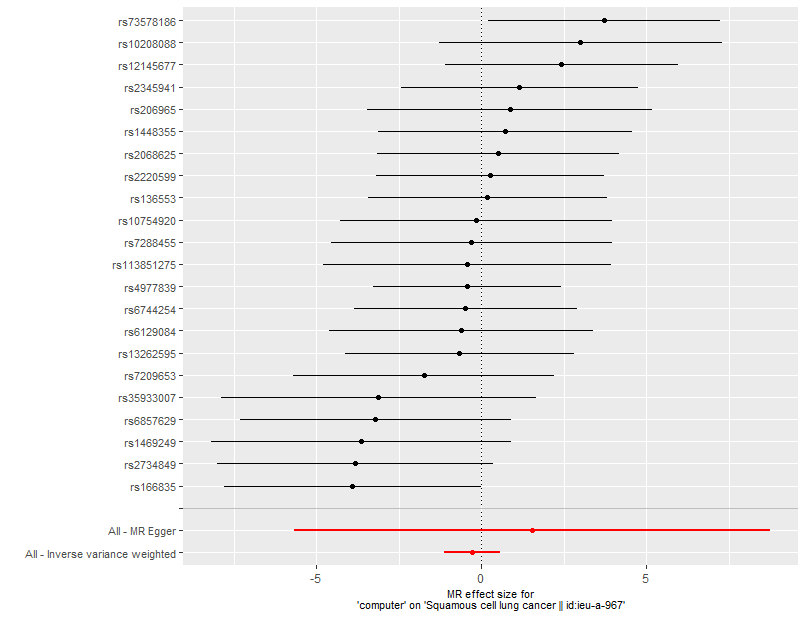


Supplementary Figure 31. Leave-one-out plot presenting the relationship between computer use and squamous cell lung cancer.


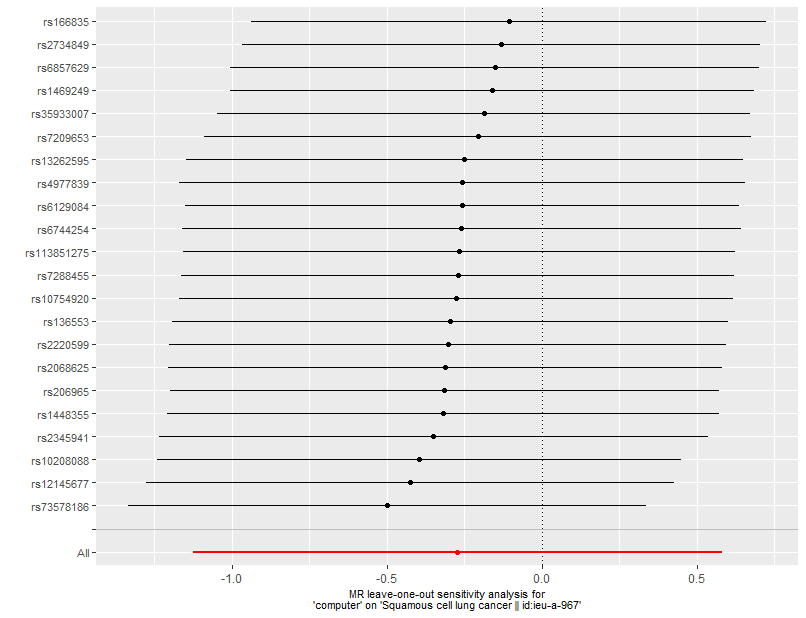


Supplementary Figure 32. Funnel plot presenting the relationship between computer use and squamous cell lung cancer.


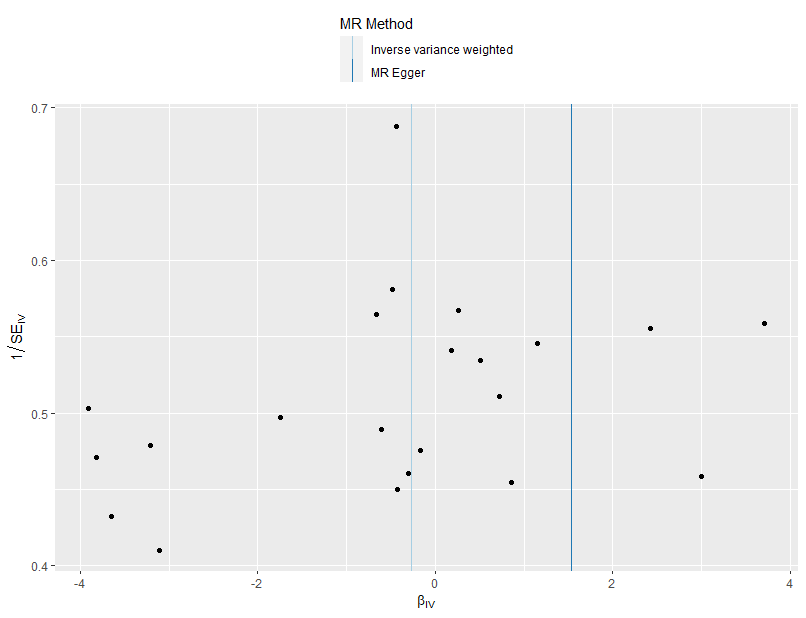


Supplementary Figure 33. Scatter plot presenting the relationship between driving and squamous cell lung cancer.


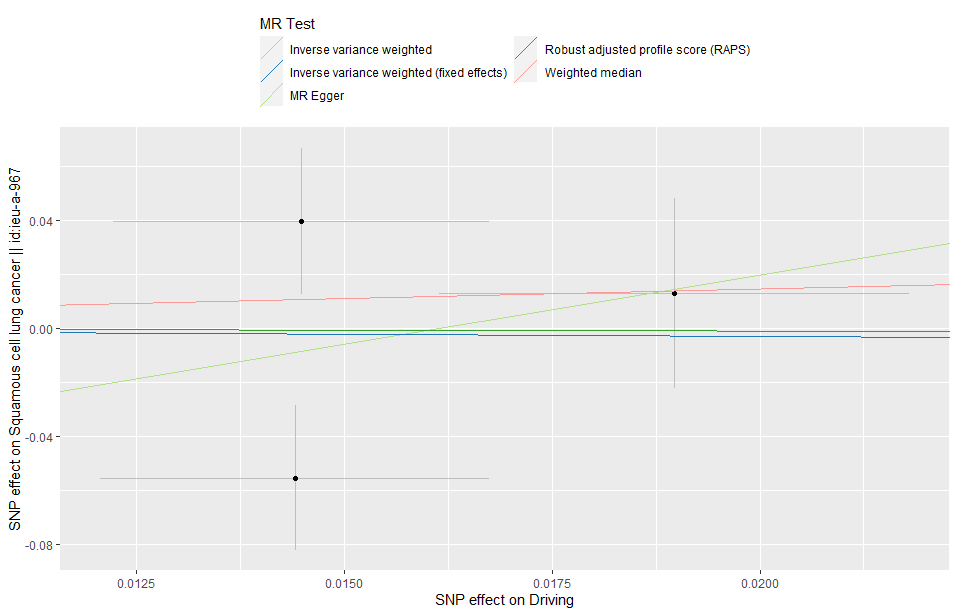


Supplementary Figure 34. Forest plot presenting the relationship between driving and squamous cell lung cancer.


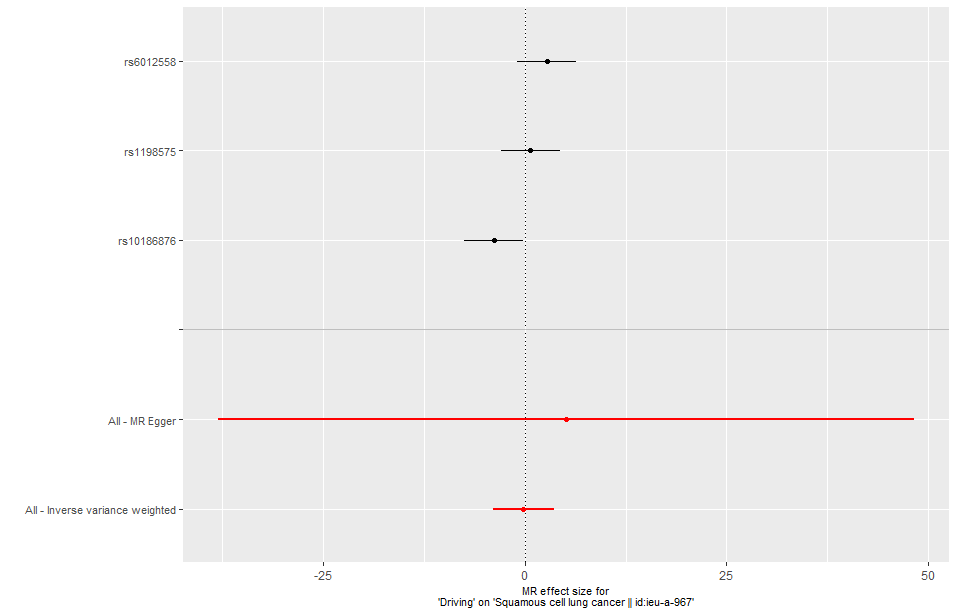


Supplementary Figure 35. Leave-one-out plot presenting the relationship between driving and squamous cell lung cancer.


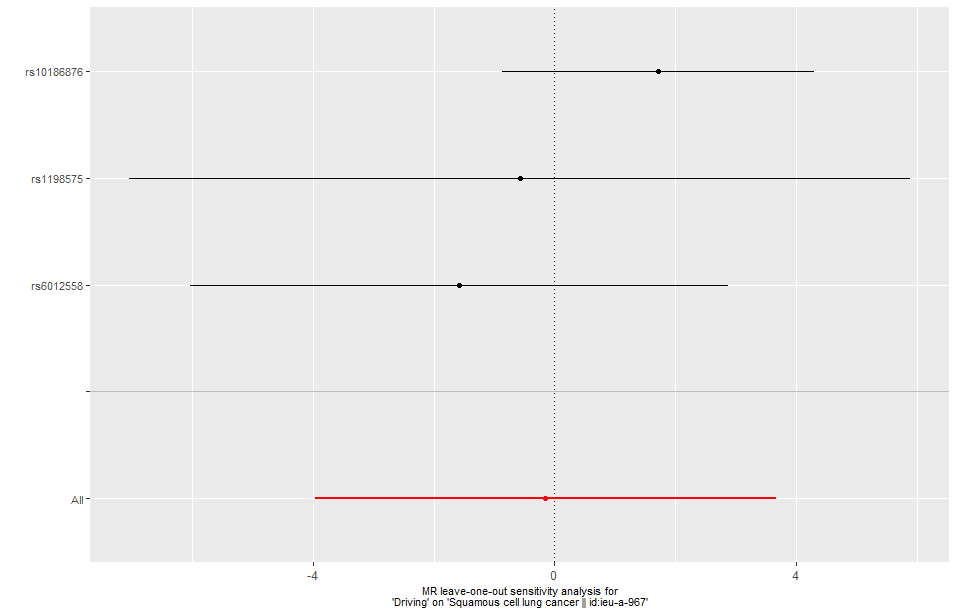


Supplementary Figure 36. Funnel plot presenting the relationship between driving and squamous cell lung cancer.


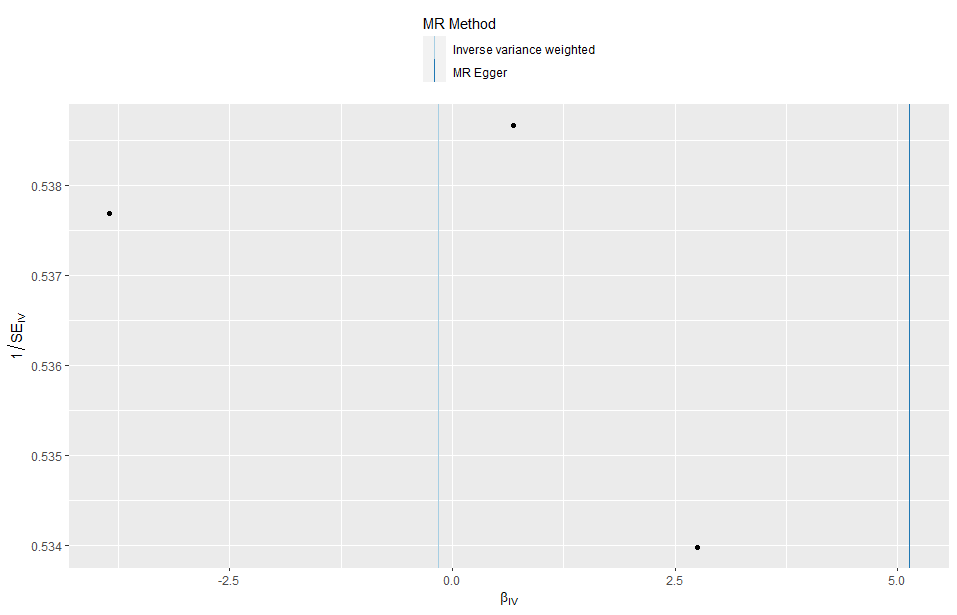

Supplement: Supplementary file 6 [file DataSheet3.DOCX]
