# Supplementary material for: Leisure Sedentary Behavior and Risk of Lung Cancer: A Two-Sample Mendelian Randomization Study and Mediation Analysis
Source: Front Genet. 2021 Oct 28;12:763626. doi: 10.3389/fgene.2021.763626 (PMC8582637; doi:10.3389/fgene.2021.763626)

Supplementary Figure 13. Scatter plots presenting the relationship between television watching and lung adenocarcinoma.


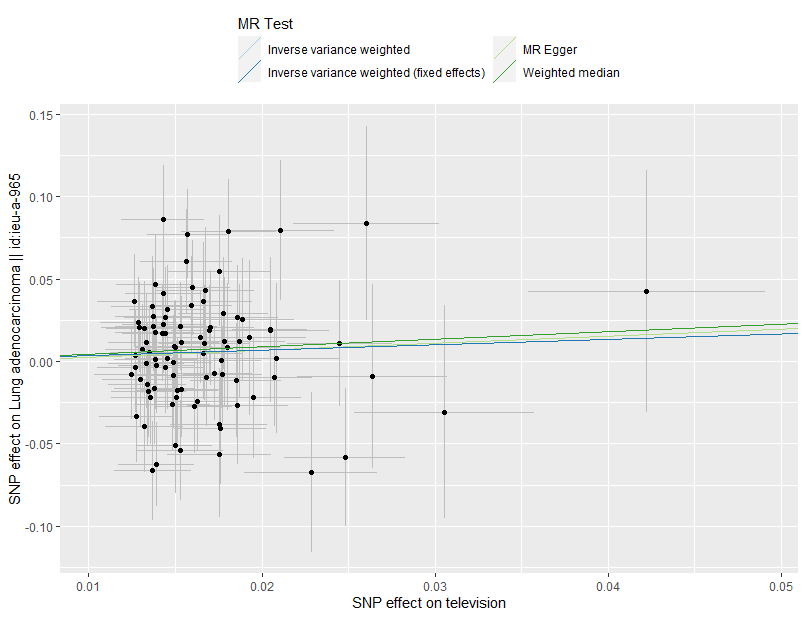


Supplementary Figure 14. Forest plots presenting the relationship between television watching and lung adenocarcinoma.


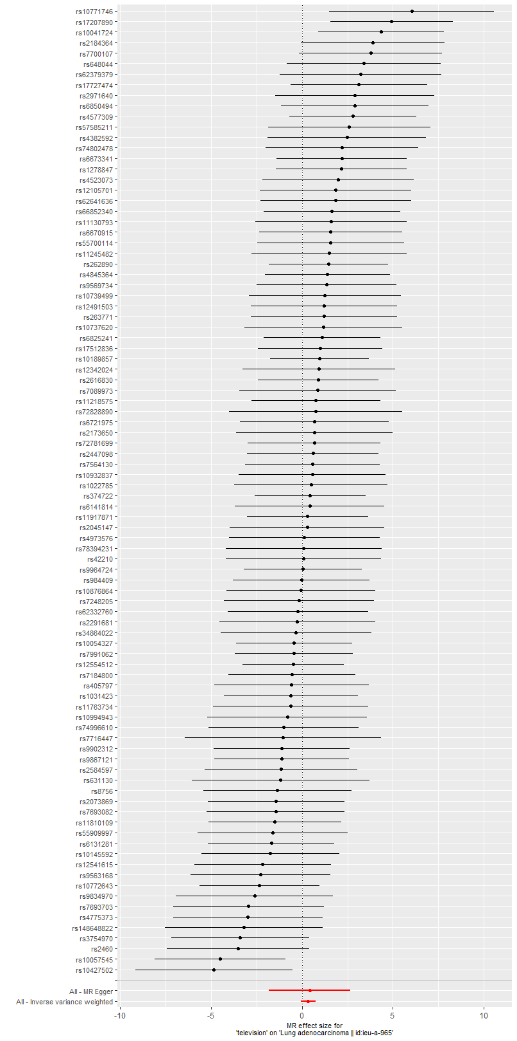


Supplementary Figure 15. Leave-one-out plot presenting the relationship between television watching and lung adenocarcinoma.


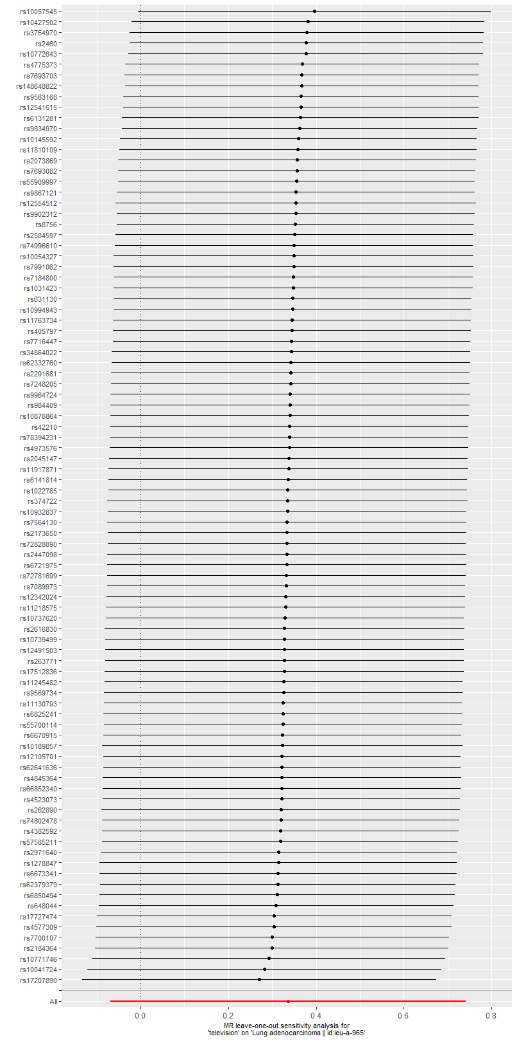


Supplementary Figure 16. Funnel plots presenting the relationship between television watching and lung adenocarcinoma.


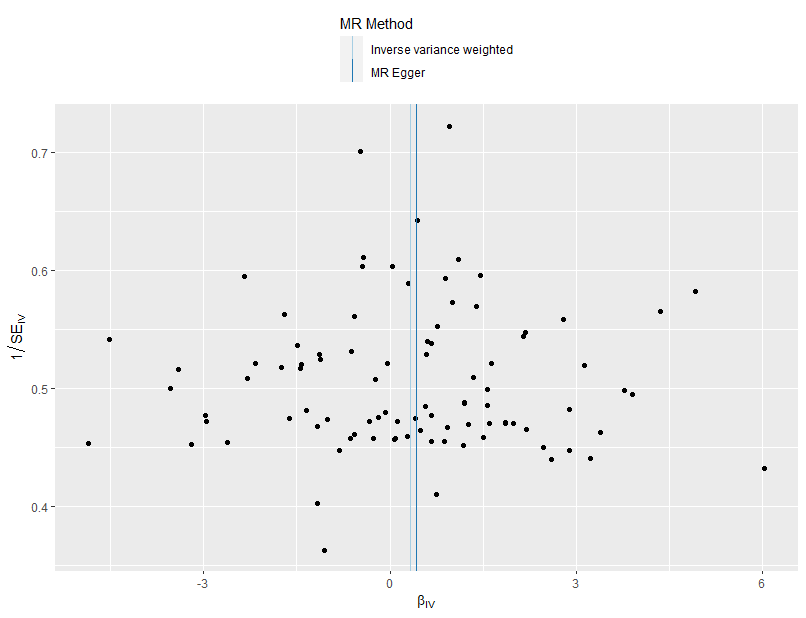


Supplementary Figure 17. Scatter plot presenting the relationship between computer use and lung adenocarcinoma.


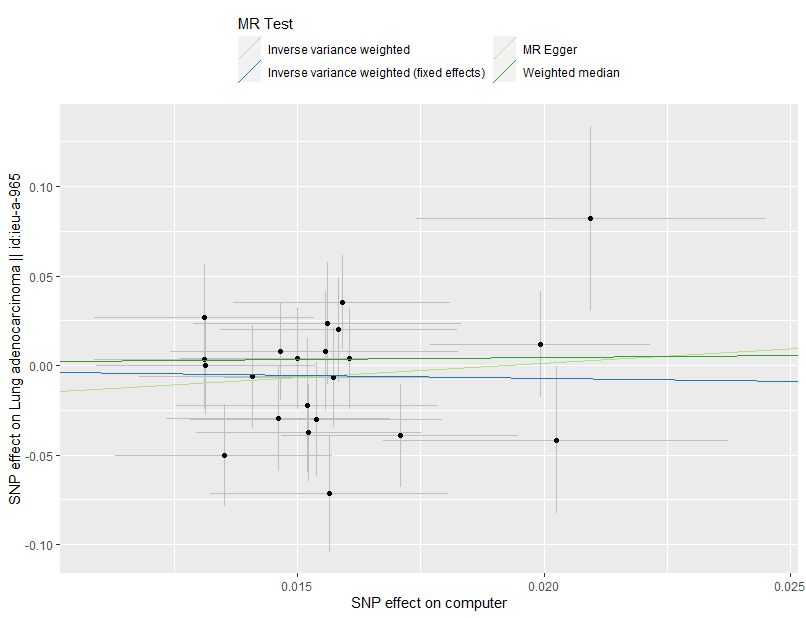


Supplementary Figure 18. Forest plot presenting the relationship between computer use and lung adenocarcinoma.


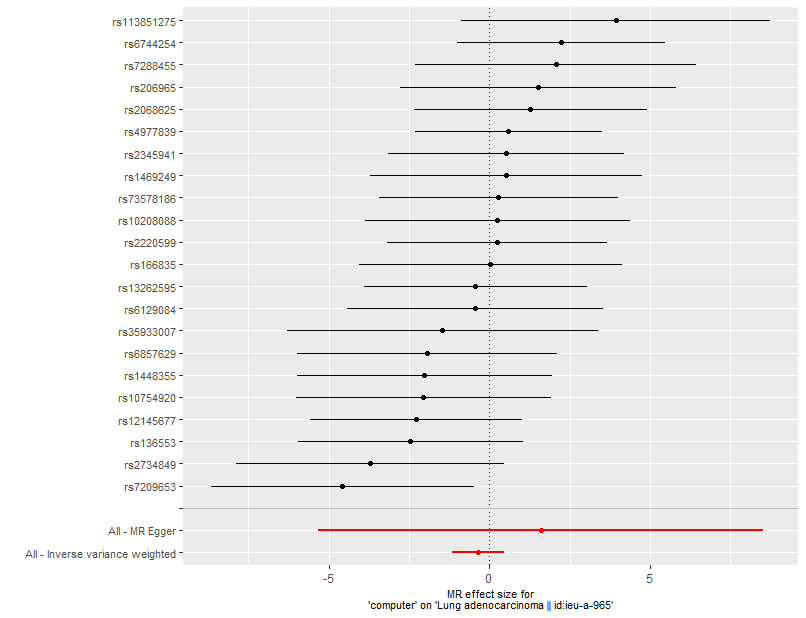


Supplementary Figure 19. Leave-one-out plot presenting the relationship between computer use and lung adenocarcinoma.


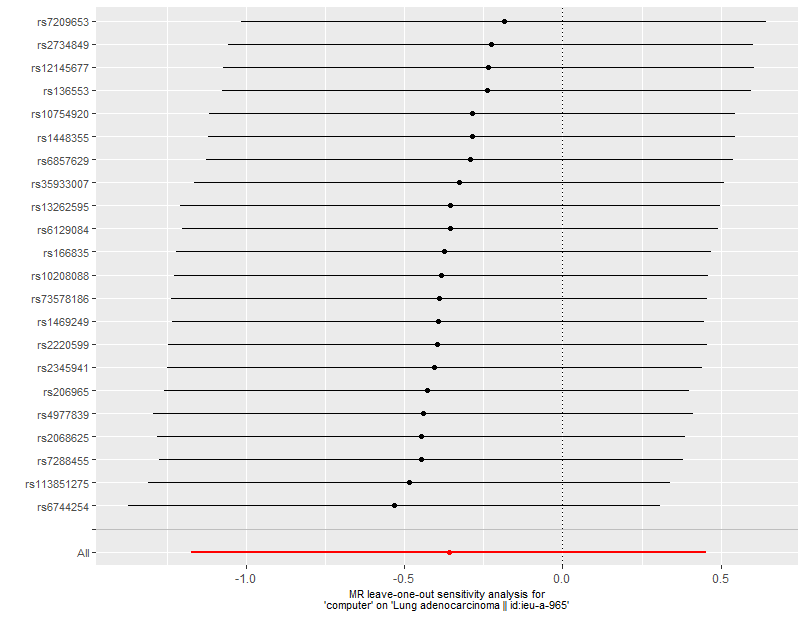


Supplementary Figure 20. Funnel plot presenting the relationship between computer use and lung adenocarcinoma.


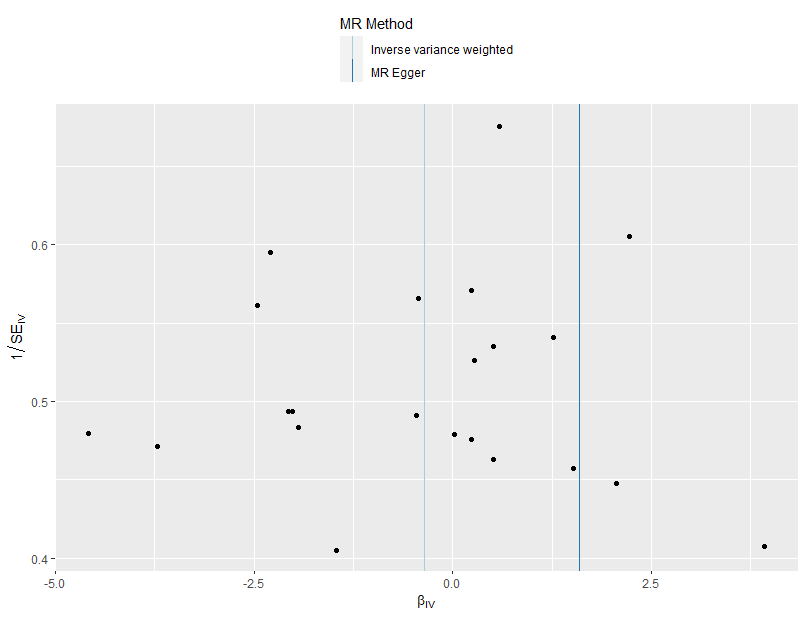


Supplementary Figure 21. Scatter plot presenting the relationship between driving and lung adenocarcinoma.


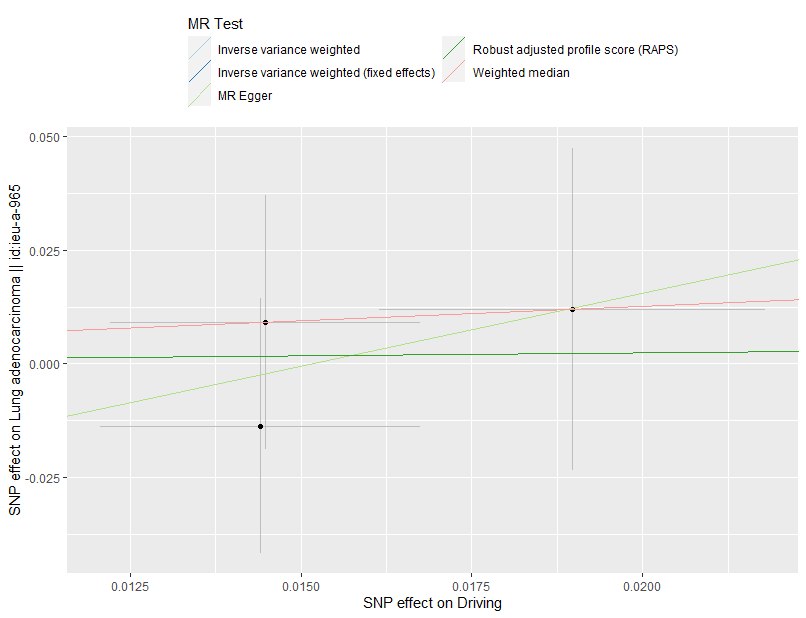


Supplementary Figure 22. Forest plot presenting the relationship between driving and lung adenocarcinoma.


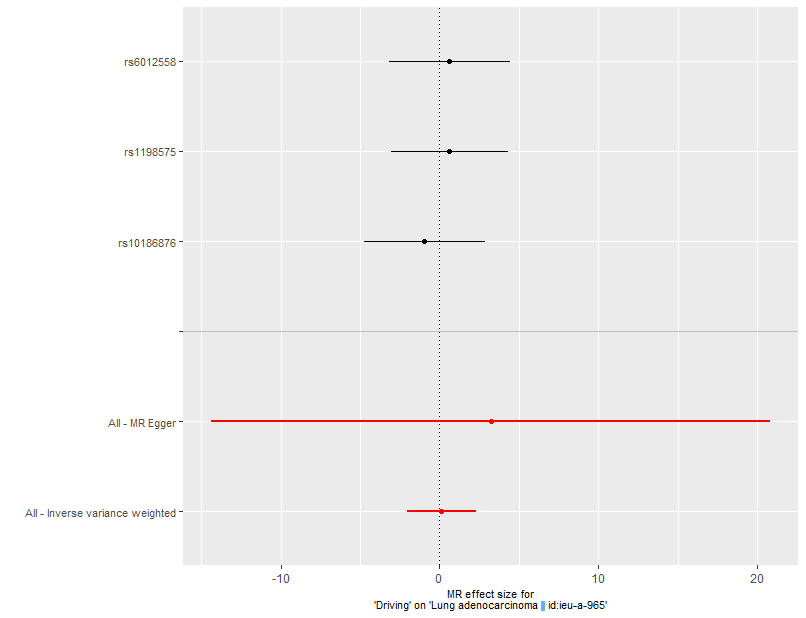


Supplementary Figure 23. Leave-one-out plot presenting the relationship between driving and lung adenocarcinoma.


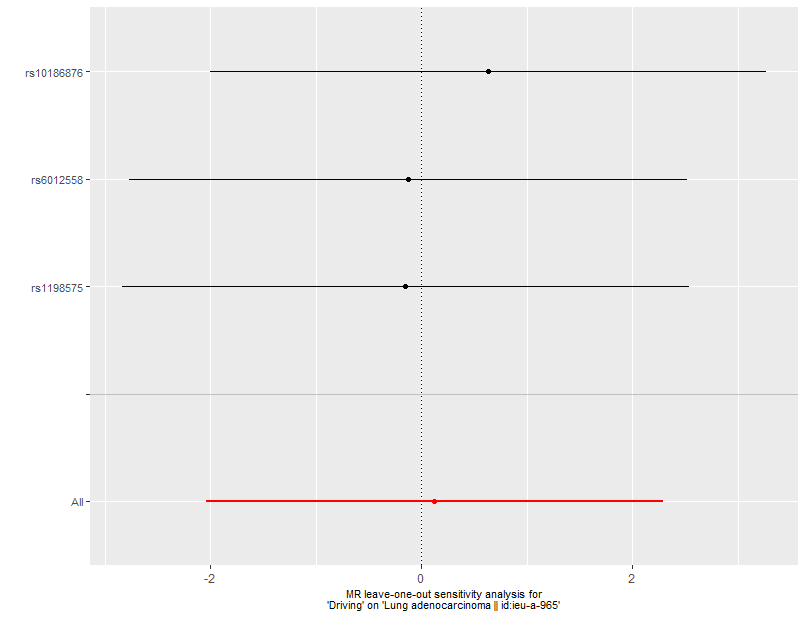


Supplementary Figure 24. Funnel plot presenting the relationship between driving and lung adenocarcinoma.


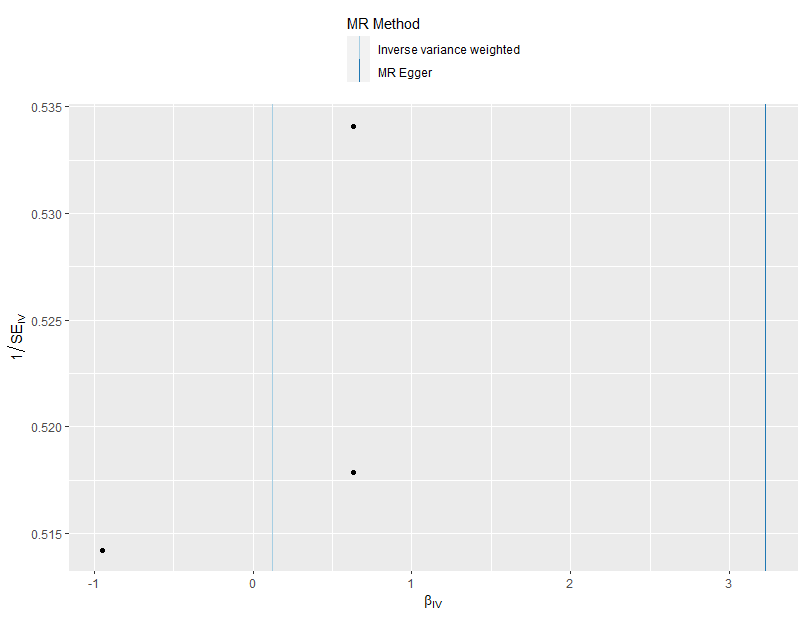

Supplement: Supplementary file 8 [file DataSheet2.DOCX]
