# Supplementary material for: Leisure Sedentary Behavior and Risk of Lung Cancer: A Two-Sample Mendelian Randomization Study and Mediation Analysis
Source: Front Genet. 2021 Oct 28;12:763626. doi: 10.3389/fgene.2021.763626 (PMC8582637; doi:10.3389/fgene.2021.763626)

Supplementary Figure 1. Scatter plots presenting the relationship between television watching and lung cancer.


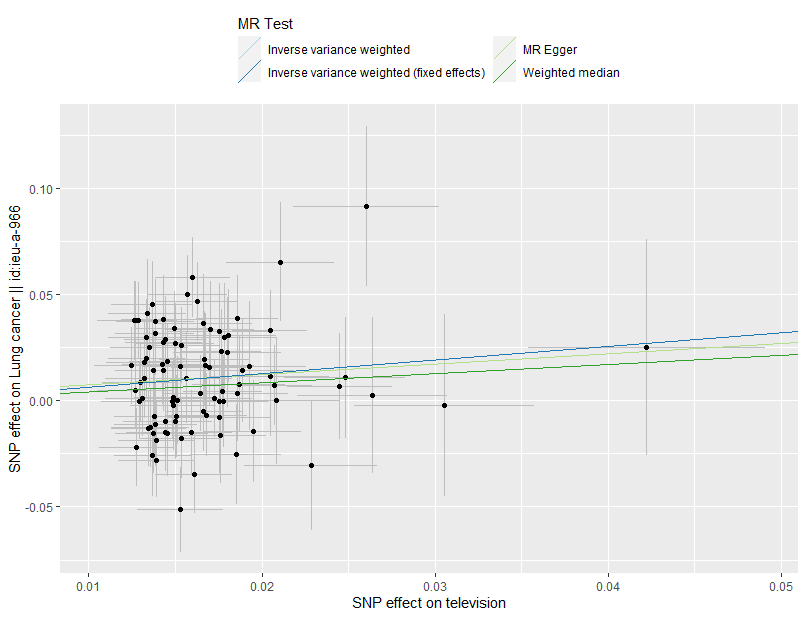


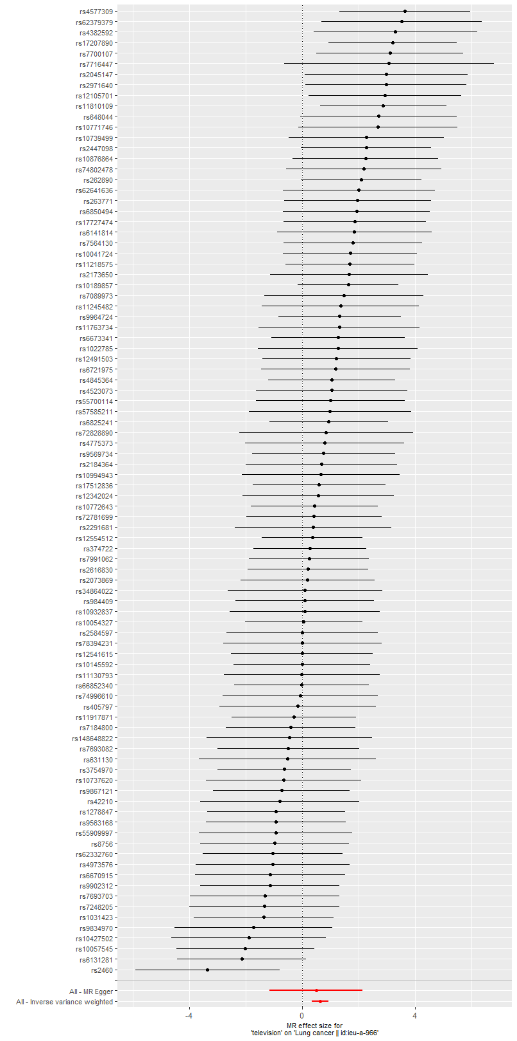
Supplementary Figure 2. Forest plots presenting the relationship between television watching and lung cancer.

Supplementary Figure 3. Leave-one-out plot presenting the relationship between television watching and lung cancer.


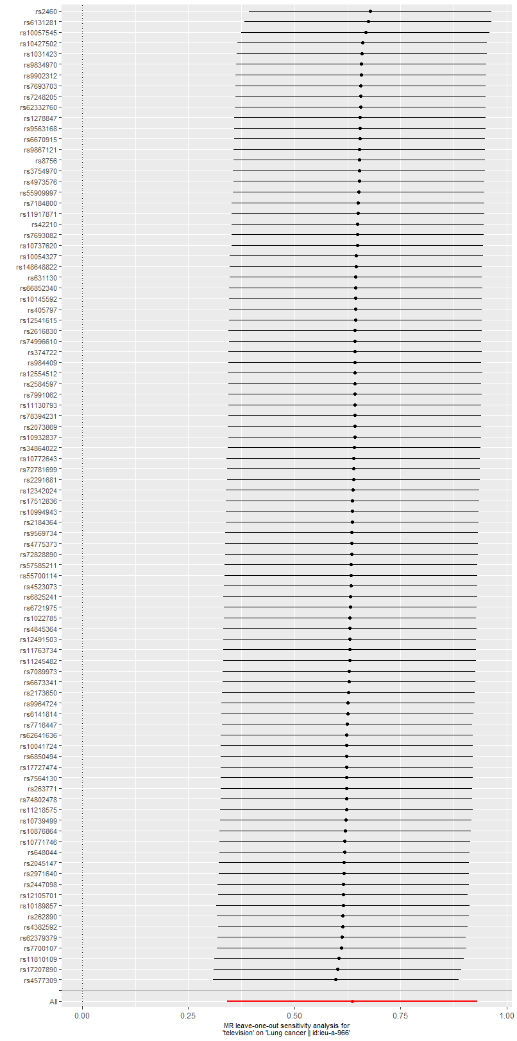


Supplementary Figure 4. Funnel plots presenting the relationship between television watching and lung cancer.


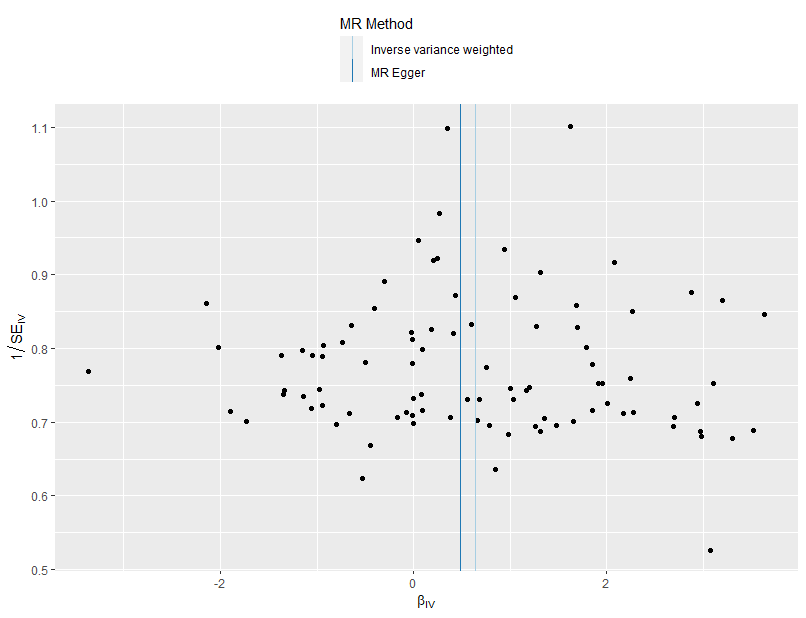


Supplementary Figure 5. Scatter plot presenting the relationship between computer use and lung cancer.


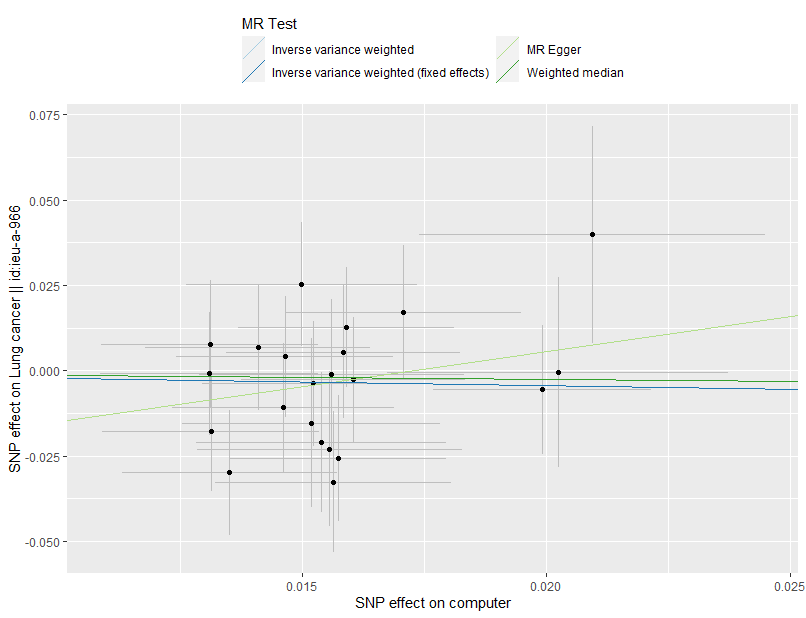


Supplementary Figure 6. Forest plot presenting the relationship between computer use and lung cancer.


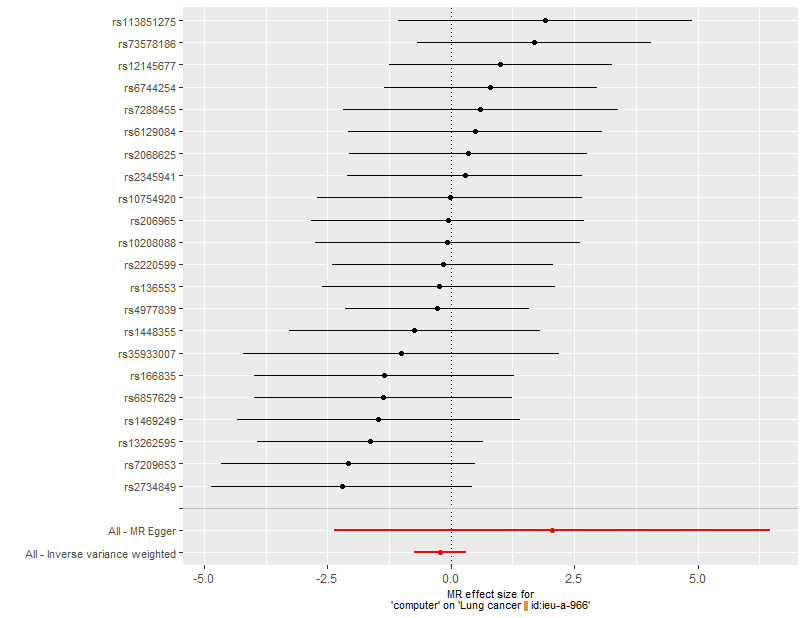


Supplementary Figure 7. Leave-one-out plot presenting the relationship between computer use and lung cancer.


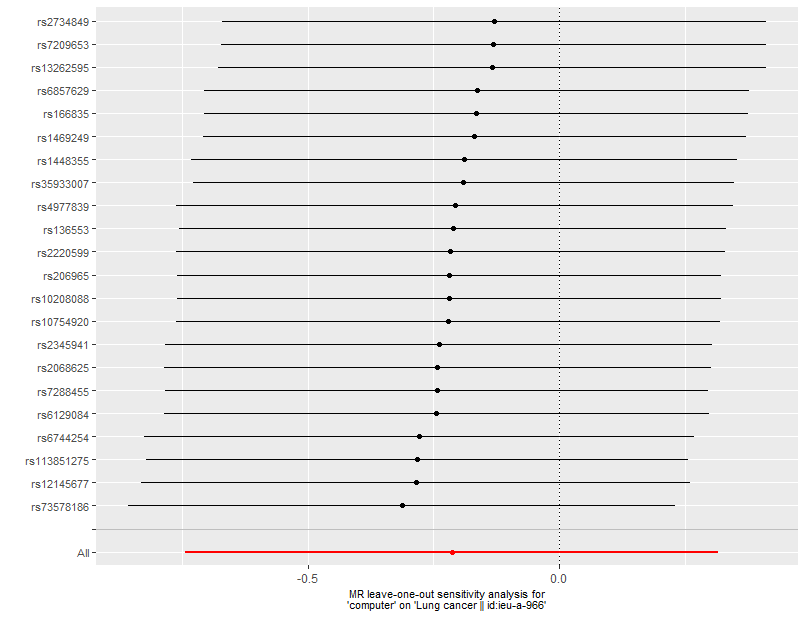


Supplementary Figure 8. Funnel plot presenting the relationship between computer use and lung cancer.


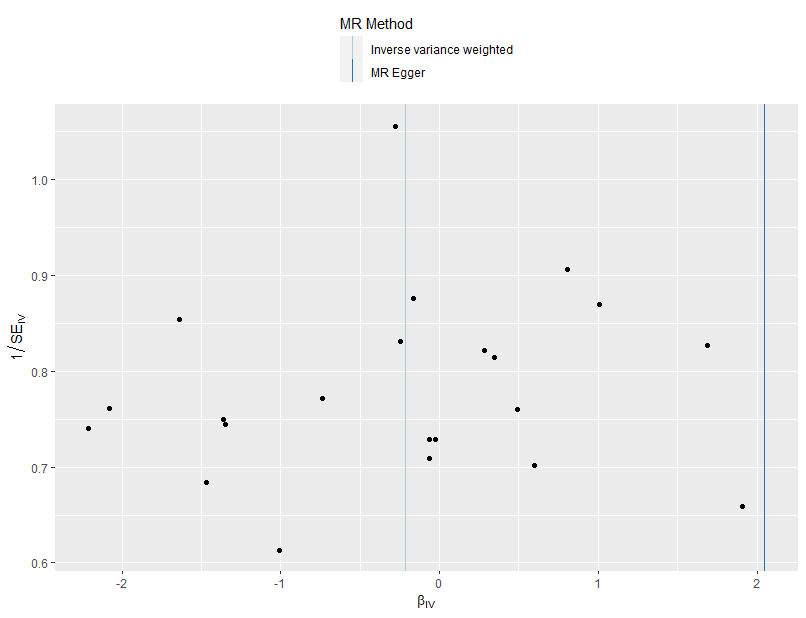


Supplementary Figure 9. Scatter plot presenting the relationship between driving and lung cancer.


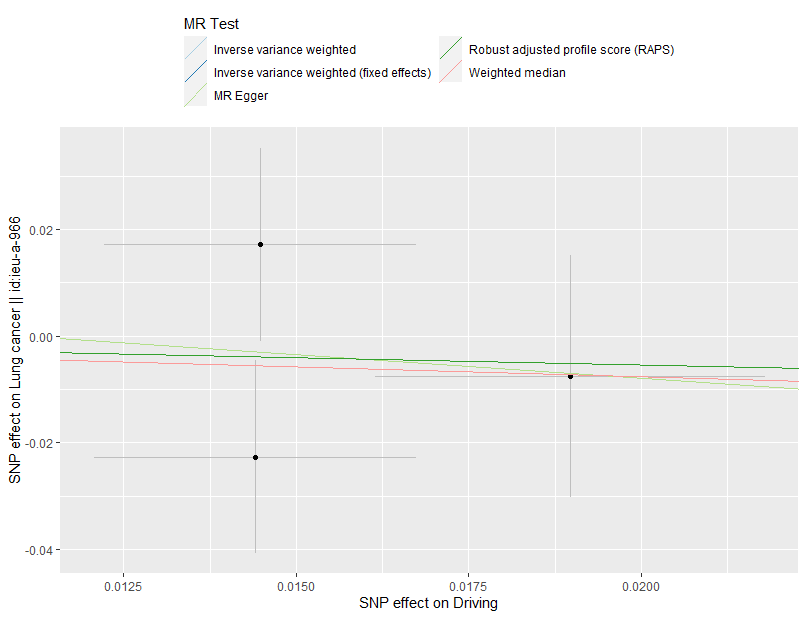


Supplementary Figure 10. Forest plot presenting the relationship between driving and lung cancer.


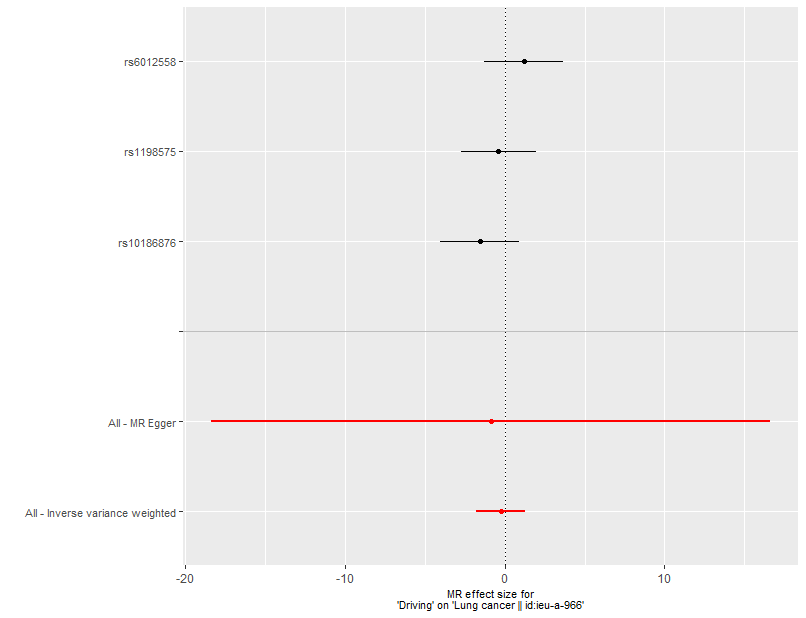


Supplementary Figure 11. Leave-one-out plot presenting the relationship between driving and lung cancer.


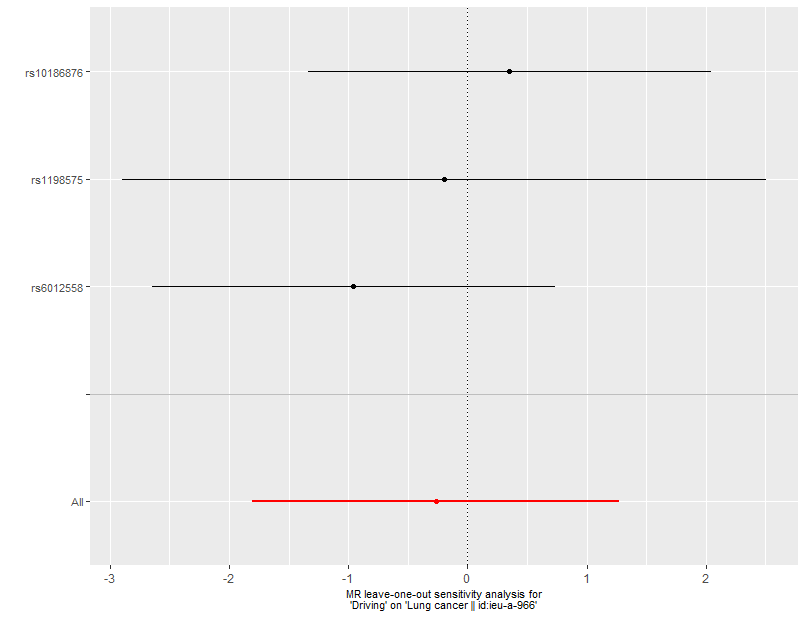


Supplementary Figure 12. Funnel plot presenting the relationship between driving and lung cancer.


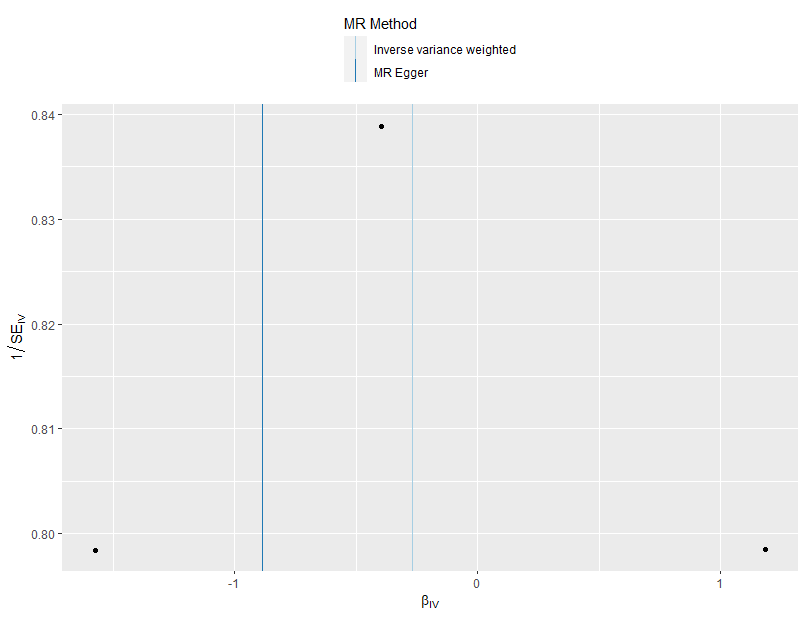

Supplement: Supplementary file 10 [file DataSheet1.DOCX]
